# Advances in the clinical management of type 2 diabetes: a brief history of the past 15 years and challenges for the future

**DOI:** 10.1186/s12916-019-1281-1

**Published:** 2019-02-26

**Authors:** Naveed Sattar

**Affiliations:** Institute of Cardiovascular and Medical Sciences, University of Glasgow, 126 University Place, Glasgow, G12 8TA UK

**Keywords:** Cardiovascular disease, Heart failure, Prevalence, Adiposity, Glycaemia, Risk factors, Remission, Prevention

## Abstract

Remarkable progress has been made in some aspects of diabetes care over the last 15 years, but there have also been a rising number of challenges that differ between high and low-income countries. In high-income countries, a substantial increase in the use of preventative drugs for cardiovascular disease has lowered vascular complications and improved diabetes survival. More recently, new classes of diabetes drugs have emerged that can variably lower cardiovascular outcomes, new-onset heart failure and slow renal decline, thereby meaningfully increasing the diabetes armoury that should help patients to live even longer lives and with fewer complications. At the other end of the disease spectrum, we can now better prevent diabetes in people who are at elevated risk of developing it, whereas other new research has shown that diabetes remission is possible when lifestyle changes are made in the early years after diagnosis. The downside is that more people than ever before have type 2 diabetes, so despite such progress in high-income countries, the absolute burden of disease is rising. Furthermore, it is rising even faster in low and middle-income countries, where rising adiposity is driving a tidal wave of new diabetes cases; yet, healthcare systems are less able to cope, lacking sufficient drugs, trained personnel and integrated care systems. Thus, despite advances, the future challenges from rising diabetes rates worldwide are daunting.

## Background

In the last 15 years we have witnessed some remarkable changes in the care of patients with type 2 diabetes. There have been many success stories in high-income countries, but also – in part arising from successes in lowering morality rates – a rising prevalence of type 2 diabetes and associated comorbidities [1]. Unfortunately, a simultaneous rise in worldwide adiposity has fuelled the rising incidence of type 2 diabetes in many low and middle-income countries, where care systems are unable to cope with their patients’ needs [2]. Consequently, the ever-increasing numbers of people with the disease – a trend that seems unlikely to slow or reverse anytime soon – presents multiple challenges.

### The emergence of intensive cardiovascular risk factor management

Around the early 2000s, the writers of diabetes guidelines in high-income countries began to recommend the comprehensive use of statins and antihypertensives. In the subsequent few years, this led to considerable improvements in related risk factors, whereas average HbA1c levels improved more modestly. Improvements in blood pressure and cholesterol levels allied to population-wide reductions in smoking rates led, in turn, to considerable improvements in cardiovascular disease (CVD) risk in type 2 diabetes, with evidence of greater reductions in CVD events in diabetes relative to the background population [1]. Notably, widespread statin use was recommended on the mistaken belief that type 2 diabetes represented a coronary heart disease risk equivalent – something we now know to be untrue, at least at the time of diagnosis. Nevertheless, many patients with type 2 diabetes were prescribed statins at somewhat lower risk thresholds than their non-diabetic counterparts; a factor contributing to greater CVD risk reductions in the diabetes population [3]. Despite our improved knowledge, most countries continue to recommend statins to all type 2 diabetes patients over the age of 40 years, with one exception: guidelines for England and Wales published by the National Institute of Health and Care Excellence have reverted to risk-scoring such patients before statins are allocated. A key question to answer in future will be whether or not cholesterol levels start to rise in English and Welsh diabetes populations, thus attenuating CVD gains.

### The advent of new diabetes drugs proven to reduce risks of cardiovascular and related outcomes

Since type 2 diabetes is diagnosed based on glycaemia levels, important changes over the last 15 years began with the emergence of multiple new anti-diabetes  therapies (DPP-4 inhibitors, GLP-1-receptor agonists, and SGLT2 inhibitors). Around the same time, intensive glucose-lowering trials failed to lower mortality or cardiovascular outcomes, and – in some cases – caused harm. Added to the rosiglitazone debacle [4], this led the US Food and Drug Administration and the European Medicines Agency to recommend all new diabetes drugs to be tested in cardiovascular outcome trials [5]. These agencies wanted pharmaceutical companies to demonstrate that their new diabetes drugs were safe from a cardiovascular perspective. A raft of cardiovascular outcome trials followed – the remarkable results of which have now substantially altered the clinical landscape.

A short summary of the outcome trials reported so far is useful. Notably, all included patients had existing cardiovascular disease or were at elevated risk, so the cardiology community was very attentive to the results. In the first three trials, all tested DPP-4 inhibitors proved to be safe from a cardiovascular perspective, although a greater risk of hospitalisation caused by heart failure was noted for saxagliptin – a finding still unexplained [6]. However, there was no evidence of CVD protection in any of these trials. The fourth trial tested lixisenatide, a short-acting GLP-1RA, in diabetes patients after an acute coronary syndrome; it was also neutral on all counts [7]. At this point, many researchers in the diabetes community began to question the wisdom of investing so much time, money and effort in such clinical trials. Some cardiologists questioned the clinical value of diabetes drugs per se, arguing that they changed only a surrogate risk marker (HbA1c), but not hard outcomes. All of these doubts diminished after investigators leading the EMPAREG Outcome trial [8] reported their results for empagliflozin in 2013 – results now broadly validated by two other SGLT2 inhibitor trials (CANVAS [9] and DECLARE [10]), and by a recent meta-analysis of all three trials [11]. Doubts further lessened with the positive cardiovascular outcome results of LEADER [12], reported in 2014, which tested liraglutide. Several other GLP-1RA trials have also since reported cardiovascular benefits [13], so that now two broad classes lessen CVD outcomes.

Of particular note are the remarkable and unexpected beneficial effects of the SGLT2 inhibitors on risks for heart failure hospitalisation, decline in estimated glomerular filtration rate and hard renal outcomes. Furthermore, that these drugs seem to offer such benefits largely independent of their glycaemic effects has helped refocus the field on novel risk pathways for these benefits, as well on providing new understanding of the complication risks in diabetes [14].

While glycaemia levels are more strongly improved with the GLP-1RA class of drugs, some novel effect of these drugs must also, in part, underlie outcome benefits. This is best hinted at by the results of the Harmony Outcomes trial [15], in which sizeable outcome benefits occurred despite the modest effects of albiglutide on established risk factors. There are, of course, important side effects with both drug classes, such as genital infections and diabetic ketoacidosis with SGLT2 inhibitors (necessitating sick day rules advice) and nausea and vomiting with GLP-1 RAs. That noted, the CVD outcome benefits of these classes, and their associated weight and blood pressure reductions and low risk for hypoglycaemia, mean that multiple clinical guidelines recommend both drug classes for use in patients with diabetes and existing CVD. Likewise, the recent consensus published jointly by the American Diabetes Association and the European Association for the Study of Diabetes recommends their wider use [16] and doctors are responding with evidence of a gradual increase in the use of these medications both in patients with diabetes and prevalent CVD, and those at elevated risk of CVD.

SGLT2 inhibitor trials have helped to shine a stronger spotlight on heart failure risks in type 2 diabetes at a time when such risks have matched – and even exceeded – those for acute myocardial infarction or strokes as first vascular events in diabetes patients. The diabetes community has thus renewed its interest in the mechanisms responsible for heart failure, with multiple new studies targeting the haemodynamic axis, in particular [17]. Further trials on the SGLT2 class of drugs in patients with existing heart failure and chronic kidney disease are ongoing and include patients with both diabetes and pre-diabetes [18–22]. If successful, new options to prevent and treat such conditions will emerge. Cardiologists and nephrologists are excited by such prospects and multiple joint meetings have been promoted between relevant specialities.

### Back to the future: getting better at – and more serious about – lifestyle interventions

Fortunately, progress in type 2 diabetes has not been restricted to greater use of drugs or new drug trial findings, but extends to an improvement in the delivery of lifestyle changes. With respect to diabetes prevention, which is cost-effective, several countries have initiated national programmes seeking people at elevated risk. How these develop and whether they make any real dent in the numbers of people developing diabetes are major questions of interest. Many doctors remain sceptical on this point and believe that only governments and legislative changes directed at the food and drink industry will make any real inroads to rising obesity levels (and so diabetes rates) worldwide. I agree.

Beyond prevention, diabetes remission is now an achievable target for many. The DiRECT trial showed that using a low-calorie diet to lose around 10 kg of weight can lead to around half the number of type 2 diabetes patients diagnosed within the last six years to no longer have diabetes one year later. The intervention involved an initial low-calorie (just over 800 kcal per day) diet phase for 3–5 months, followed by food reintroduction and a weight maintenance phase. It was cost-effective and patients’ quality of life improved [23, 24]. The parallel mechanistic insights pointed to striking improvements in liver fat levels, with weight loss and evidence of β-cell recovery in the subset undergoing remission, thus tying weight loss to distinct benefits in the diabetes pathway [25]. These trial results have led the National Health Service in England to pilot this intervention more widely in clinical practice. Several other countries are also undertaking similar trials in their populations, since the greatest wish for many diabetes patients is to take fewer drugs and be free from their type 2 diabetes label [26]. While this approach is exciting, it is clear that more work is needed to help people maintain their initial weight loss.

## Conclusions

There have been multiple changes in the care of people with type 2 diabetes over the last 15 years, with much to be optimistic about given the development of better ways to prevent, reverse or treat diabetes and thus avoid its major complications. That noted, the biggest challenge remains the increasing number of people worldwide who are overweight or obese, leading to an inevitable rise in type 2 diabetes rates in nearly all countries. Yet, I and many others believe that the obesity epidemic cannot be solved by the medical profession but only by governments. However, few, if any, national governments appear to take these issues seriously. The increasing number of younger people with type 2 diabetes is a particular cause for concern because the condition worsens more rapidly in younger people, leading to greater life years lost. In high-income countries, the other challenge that paradoxically arises from our improved ability to prevent premature deaths from CVD, cancer and other chronic conditions, is that more people with such conditions are living longer and thus able to develop type 2 diabetes. At the same time, improved survival in diabetes per se gives these patients more time to develop other chronic conditions. The consequence is a rise in the number of people with comorbidity – an issue set to add to the complexities of diabetes care in the future. Finally, the rapid rise in diabetes rates in many low and middle-income countries challenges many health systems worldwide. Sadly, a lack of resources, including drugs, plus too few trained healthcare professionals and a lack of integrated care systems, means that diabetes is set to become a far more common cause of morbidity and mortality in many countries.

## Abbreviation

CVD: Cardiovascular disease
